# Supplementary material for: Carbonized paramagnetic complexes of Mn (II) as contrast agents for precise magnetic resonance imaging of sub-millimeter-sized orthotopic tumors
Source: Nat Commun. 2022 Apr 11;13:1938. doi: 10.1038/s41467-022-29586-w (PMC9001709; doi:10.1038/s41467-022-29586-w)
Supplement: Supplementary file 1 — Supplementary Information [file 41467_2022_29586_MOESM1_ESM.pdf]

Supporting Information for

Carbonized paramagnetic complexes of Mn (II) as contrast agents for precise magnetic resonance imaging of sub-millimeter-sized orthotopic tumors

Ruixue Qin[1], Shi Li[1], Yuwei Qiu[1], Yushuo Feng, Yaqing Liu, Dandan Ding, Lihua Xu, Xiaoqian Ma, Wenjing Sun, Hongmin Chen*

State Key Laboratory of Molecular Vaccinology and Molecular Diagnostics & Center for Molecular Imaging and Translational Medicine, School of Public Health, Xiamen University, Xiamen 361102, China

*Corresponding author:

hchen@xmu.edu.cn (H.C.)

[1]These authors contributed equally to this work.

Supporting Experimental Section

Materials and Characterization

4-nitrophenyl chloroformate 3-(4,5-dimethylthiazol-2-yl)-2,5-diphenyltetrazolium bromide (MTT) were purchased from Sigma-Aldrich (MO, USA). Dialysis membrane, standard, RC, 3 KD was purchased from BBI. Coverslip/Coverglass was purchased from Warner Instruments Inc. Rodent tail vein catheter was purchased from Braintree Scientific, Inc. U87 MG-RFP, horse serum were obtained from Shanghai Zhong Qiao Xin Zhou Biotechnology Co. Ltd. Dextran Texas Red was purchased from Thermo Fisher Scientific. CUBIC reagent (solution 1, 2 and 3) was purchased from Nuohai Life Science Co, Ltd. Coomassie brilliant bule 250 was purchased from Sangon Biotech (Shanghai) Co., Ltd. We are grateful to the First Affiliated Hospital of Xiamen University for providing Magnevist (Gd-DTPA) (Bayer Schering Pharma AG).

Transmission electron microscopy (TEM) images were obtained by a FEI Tecnai G2 F30 Twin transmission electron microscope with a working voltage of 300 kV. X-ray diffraction (XRD) pattern was obtained using Bruker D8 Advance X-ray diffractometer. X-ray photoelectron spectroscopy (XPS) analyses were carried out with a K-Alpha+ X-ray Photoelectron Spectroscopy using a monochromatic Al Kα source (6 mA, 12 kV) (Thermo fisher Scientific). The Raman spectrum was recorded using a laser RM2000 laser Raman spectrometer by excitation of 785 nm. The Fourier transform infrared spectrum was obtained on a Nicolet is10 Infrared spectrometer. Dynamic light scattering (DLS) and zeta potential were measured using Zetasizer Nano

particle analyser series (Malvern Ltd., UK). Thermogravimetric analysis (TGA) was performed by SDT Q600 (TA Instruments Co). The UV-vis absorption spectra were collected on a cary60 PC spectrophotometer. The fluorescence spectra were measured on an F-4600 spectrofluorometer with. The fluorescence images of cells were imaged using an Olympus confocal microscope (Leica Microsystems, Germany). The in vivo and ex vivo fluorescence imaging was conducted by IVIS Lumina II in vivo imaging system. The T_1-weighted MRI was performed on a 9.4 T BioSpec MRI (Bruker, Germany). Flow cytometry data were collected using Quanteon/ACEA (ACEA Biosciences) and the equipped NovoExpress for windows (version 1.5.6). Evaluation in vivo the BBB-crossing ability of Mn@CCs were acquired using Olympus FVMPE-RS multiphoton laser scanning microscope. 3D fluorescent imaging of above clearing tissue were acquired with Nuohai LS18 light sheet microscopy (Nuohai Life Science (Shanghai) Co., Ltd, laser lines: 405, 488, 561, 637 nm) with a 1×/0.25NA objective (Olympus MVPLAPO). Nobelium v1.0.4 is the data acquisition software of Nuohai LS18 light sheet microscopy, and Combine V1.0.5 is the data preprocessing software.

Supporting Figures

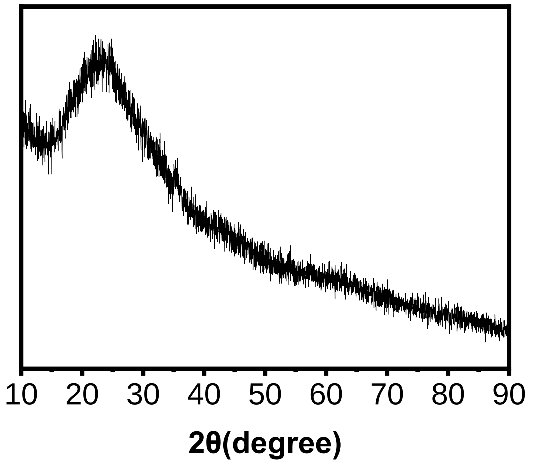

Supplementary Fig. 1. XRD pattern of Mn@CCs. The peak at around 23° (*2θ*) attributes to the weak crystallinity of graphene.

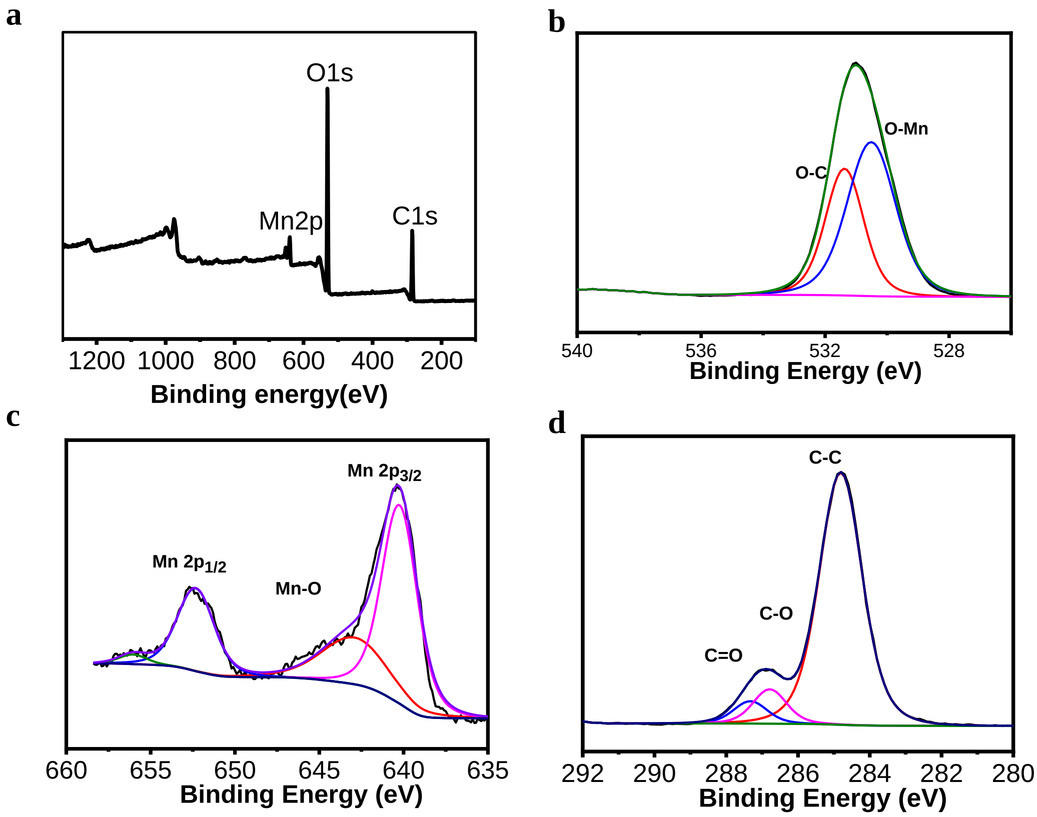

Supplementary Fig. 2. XPS spectra of the Glucose-Mn. (a) The full-scan XPS spectrum of Glucose-Mn. High-resolution XPS spectra of (b) O1s, (c) Mn2p, and (d) C1s.

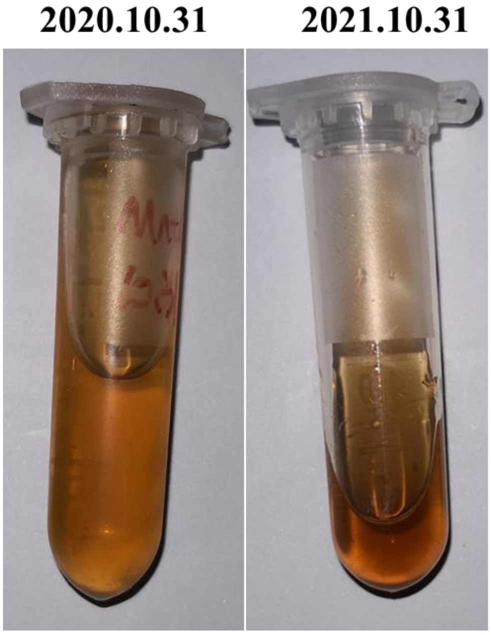

Supplementary Fig. 3. Stability of Mn@CCs. After stored at 4 ºC for a whole year (2020.10.31-2021.10.31), the solution is still a clear and transparent solution.

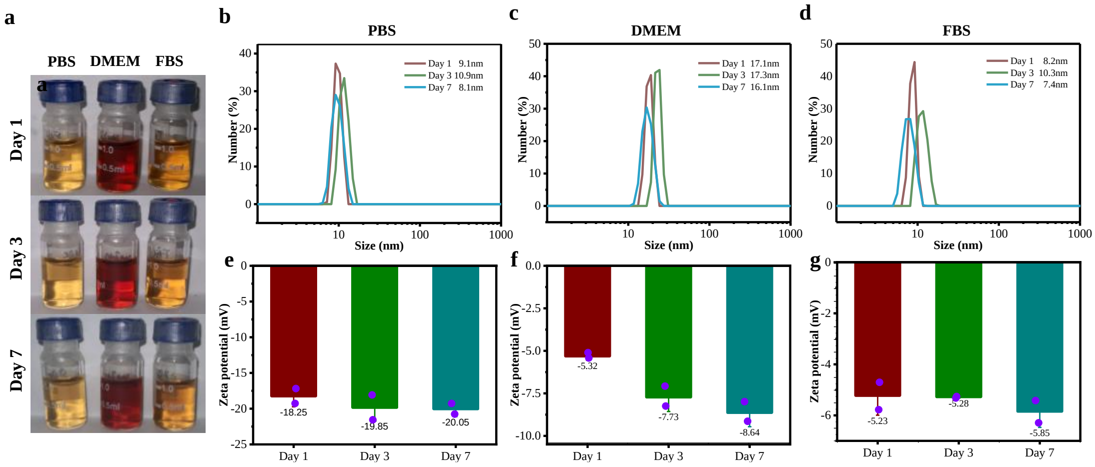

Supplementary Fig. 4. The colloidal stability of Mn@CCs. (a) Stability of Mn@CCs in different media, including PBS (pH 7.4), DMEM, and 10% FBS. (b-d) Size of Mn@CCs in different media at different time points. (e-g) Zeta potential of Mn@CCs in different media at different time points (n=2 independent experiments). All the statistical data are expressed as mean values±SD.

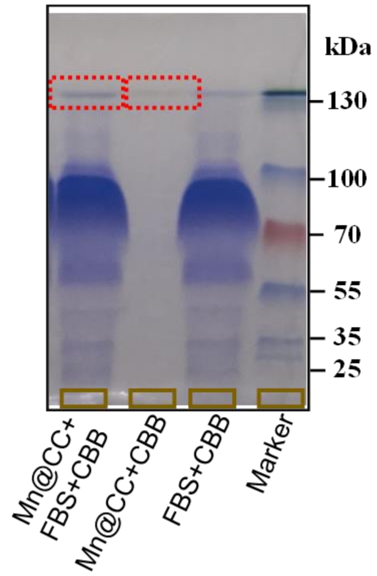

Supplementary Fig. 5. Serum protein binding test by polyacrylamide electrophoresis of Mn@CCs in PBS solution supplemented with 10% (v/v) FBS (fetal bovine serum) for 30 min under room temperature. CBB (coomassie brilliant blue 250) was used to label FBS. The result clearly shows that the Mn@CCs have little affinity to serum protein. Experiments were repeated three times.

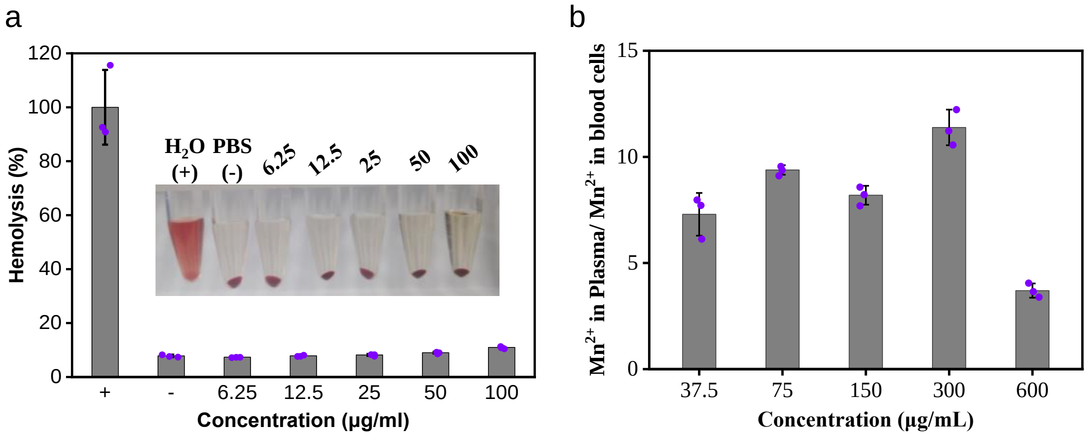

Supplementary Fig. 6. The interference between Mn@CCs and blood cells. (a) The interaction between Mn@CCs and blood cells was demonstrated by hemolysis test (n=3 biologically independent samples). All the statistical data are expressed as mean values±SD. (b) Blood-cell binding test of Mn@CCs in different concentrations (37.5, 75, 150, 300, and 600 μg/mL) (n=3 biologically independent samples). The ratio of the Mn^{2+} in the plasma to the Mn^{2+} in blood cells. All the statistical data are expressed as mean values±SD.

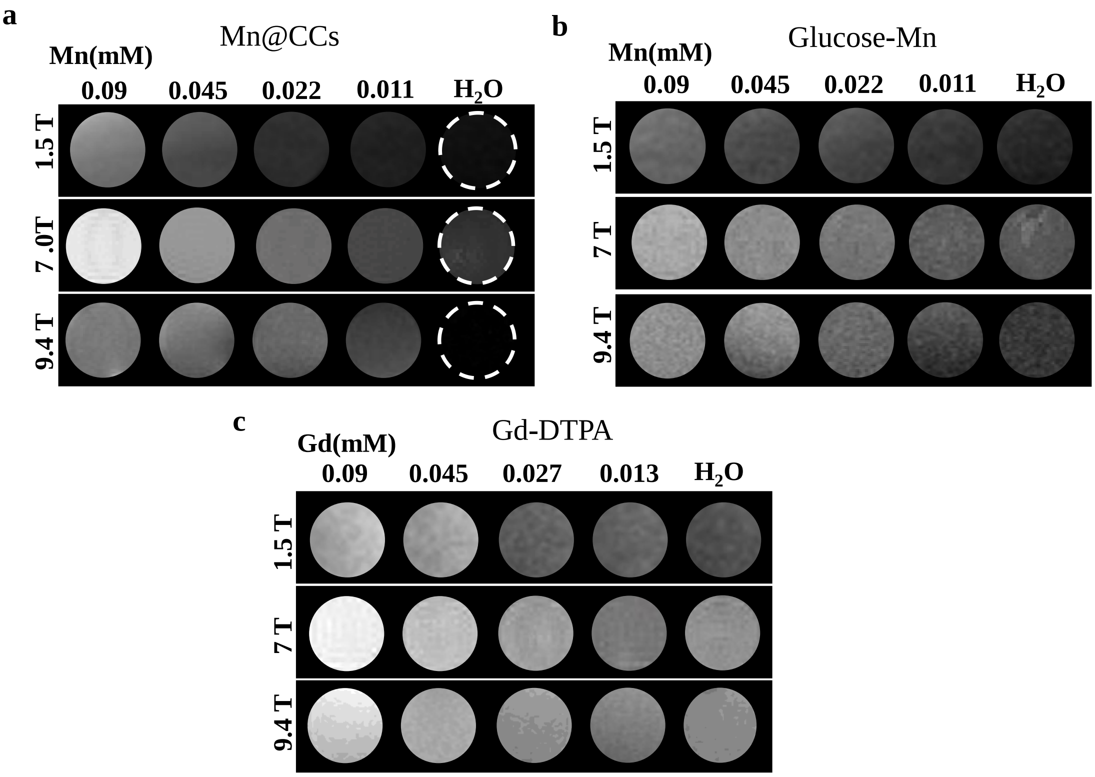

Supplementary Fig. 7. T_1 MR images of Mn@CCs (a), Glucose-Mn (b), and Magnevist (Gd-DTPA) (c) in aqueous solution investigated on the 1.5 T, 7 T and 9.4 T MRI system.

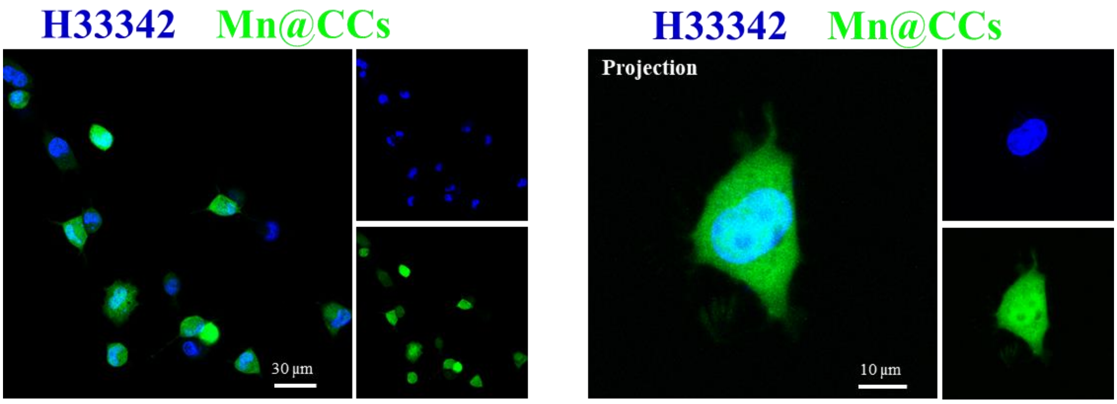

Supplementary Fig. 8. The uptake of Mn@CCs to U87MG cells by Olympus FVMPE-RS. Experiments were repeated three times. Mn@CCs: green signal, H33342: blue signal.

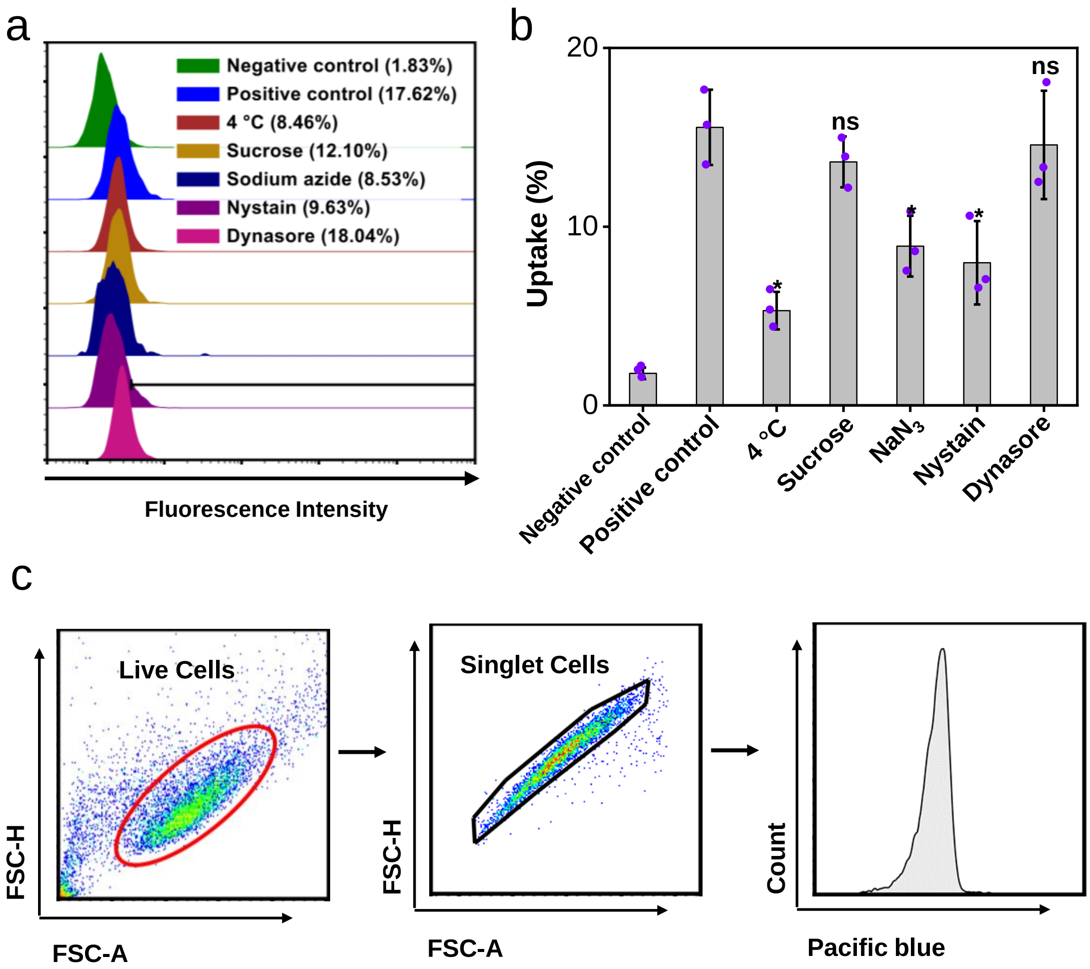

Supplementary Fig. 9. Mechanism analysis in the internalization of Mn@CCs. (a) Flow cytometry histogram analysis. (b) The corresponding proportion analysis for the uptake of Mn@CCs (n=3 independent experiments). *Significant differences from the positive control, $P < 0.05$. ns: non-significant differences from the positive control. All the statistical data are expressed as mean values± SD. Statistical significance was assessed via a one-way ANOVA with Duncan post-hoc test. (c) Gating strategies for the cell experiments in (a).

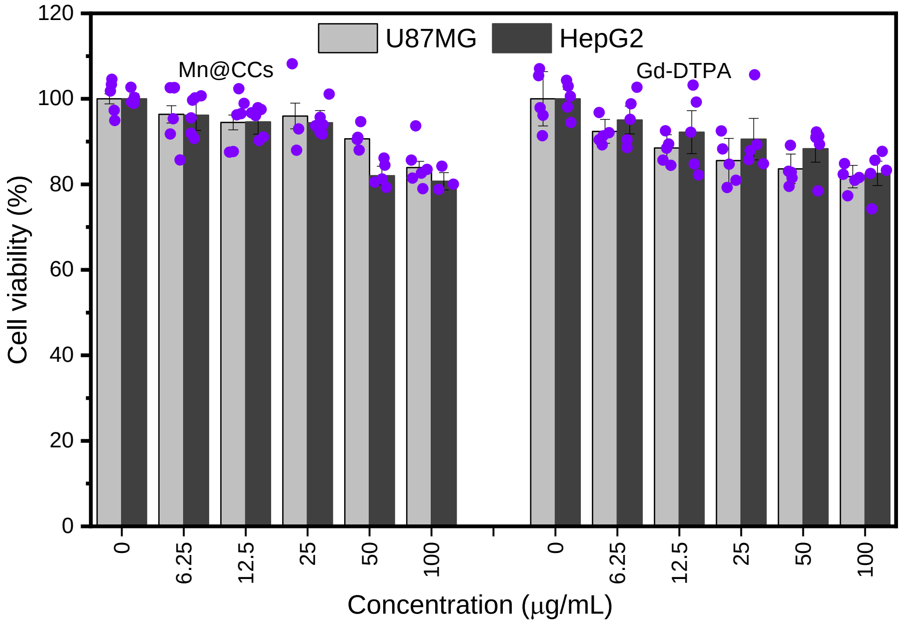

Supplementary Fig. 10. Cell viabilities evaluated by MTT assays of U87MG and HepG2 cells incubated with Mn@CCs and Gd-DTPA for 24 h. For U87MG cells incubated with Mn@CCs, 0 μg/mL n=5, 6.25 μg/mL n=6, 12.5 μg/mL n=6, 25 μg/mL n=3, 50 μg/mL n=4 and 100 μg/mL n=6. For HepG2 cells incubated with Mn@CCs: 0 μg/ mL n=4, 6.25 μg/mL n=6, 12.5 μg/mL n=6, 25 μg/mL n=6, 50 μg/mL n=5 and 100 μg/mL n=3. All experiments for cells incubated with Gd-DTPA n=5. All the statistical data are expressed as mean values±SD.

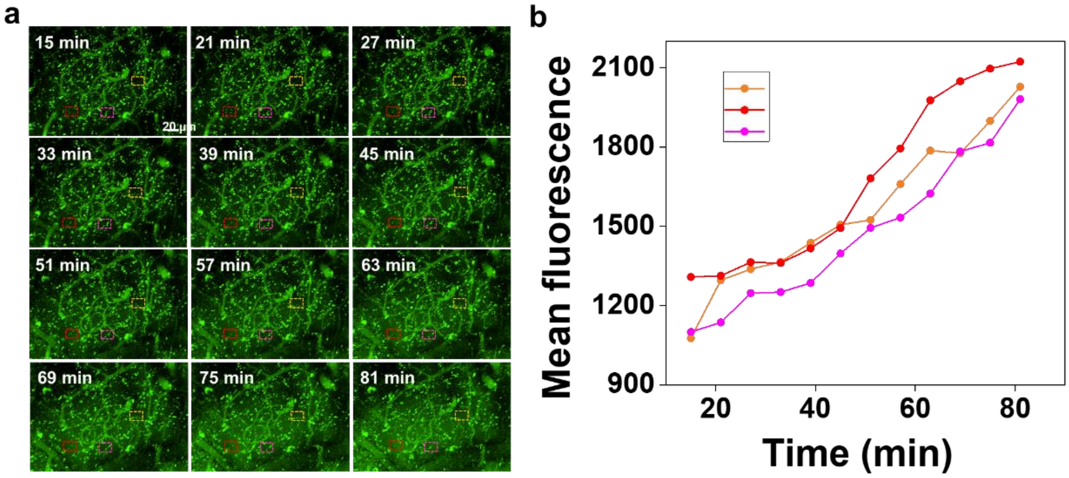

Supplementary Fig. 11. Sequential images of Mn@CCs crossing BBB on non-tumor-bearing mice (n=3 biologically independent animals). A pre-programmed program was executed every 6 min, taking 22 images in succession once time. The entire procedure was cycled 12 times for a total time of 81 min, and the first image of each circle was selected (Scale bar: 20 μm).

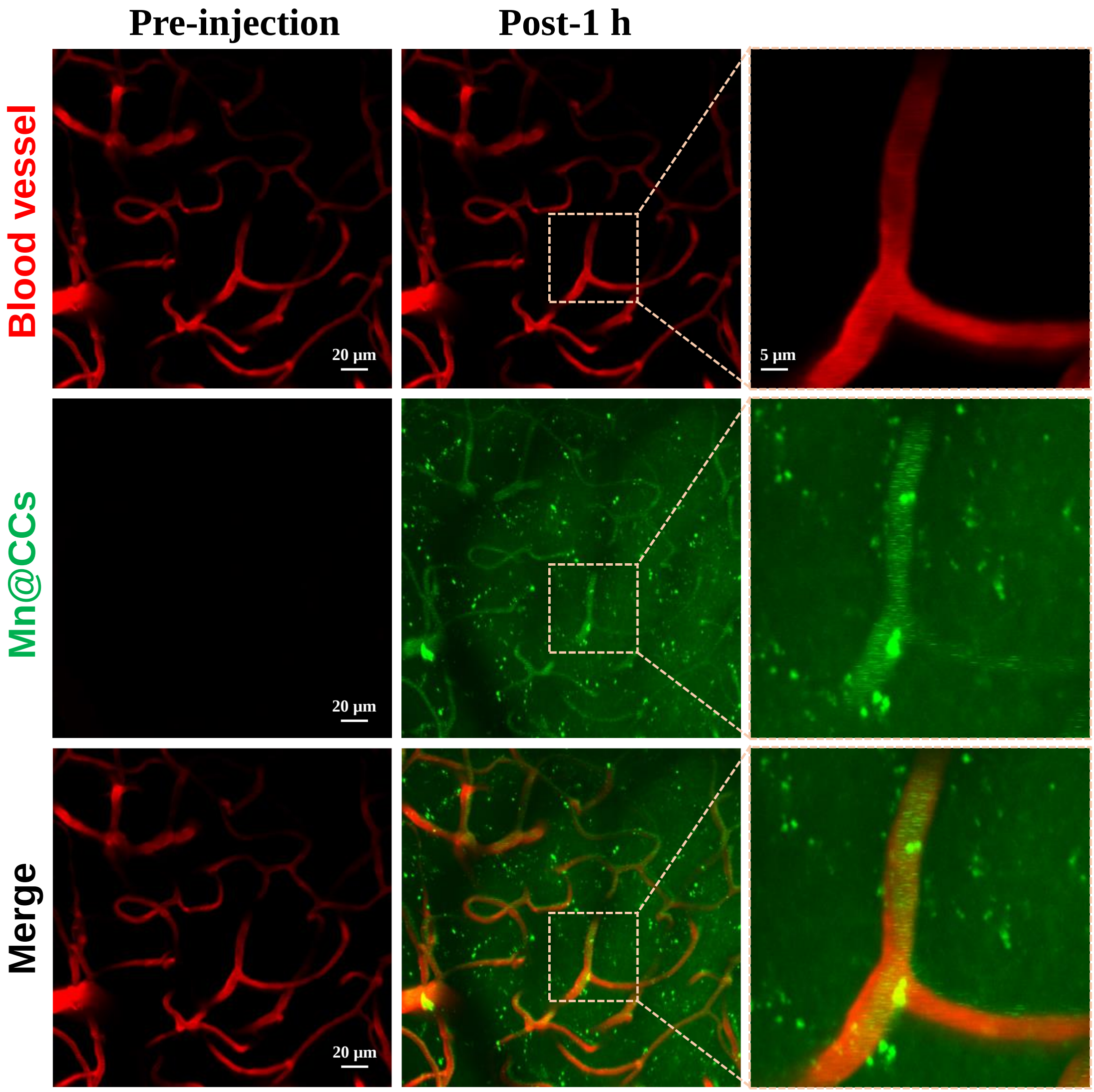

Supplementary Fig. 12. Intravital multiphoton imaging of brain of normal mice (n=3 biologically independent animals), showing diffusion of Mn@CCs (green signal) crossing BBB on normal mice. Texas Red (red signal) were pre-injected to label vasculature. Experiments were repeated three times.

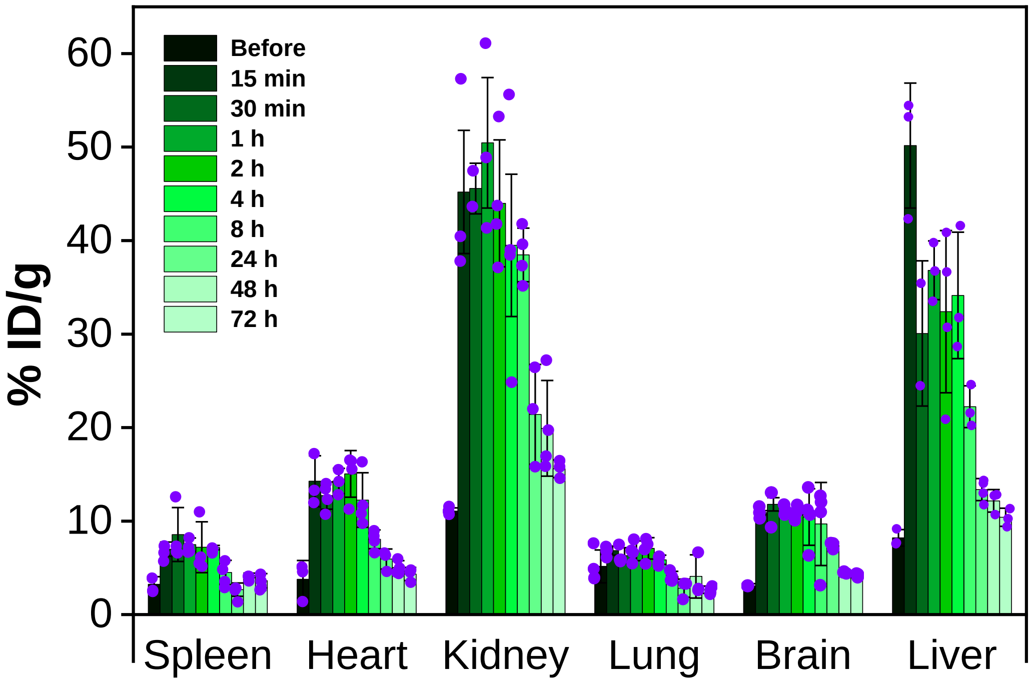

Supplementary Fig. 13. Biodistribution of Mn@CCs (Mn^{2+}) in main tissues after intravenous (i.v.) administration for varied time intervals (before, 15 min, 30 min, 1, 2, 4, 8, 24, 48 and 72 h) (4 biologically independent samples were used). All the statistical data are expressed as mean values±SD.

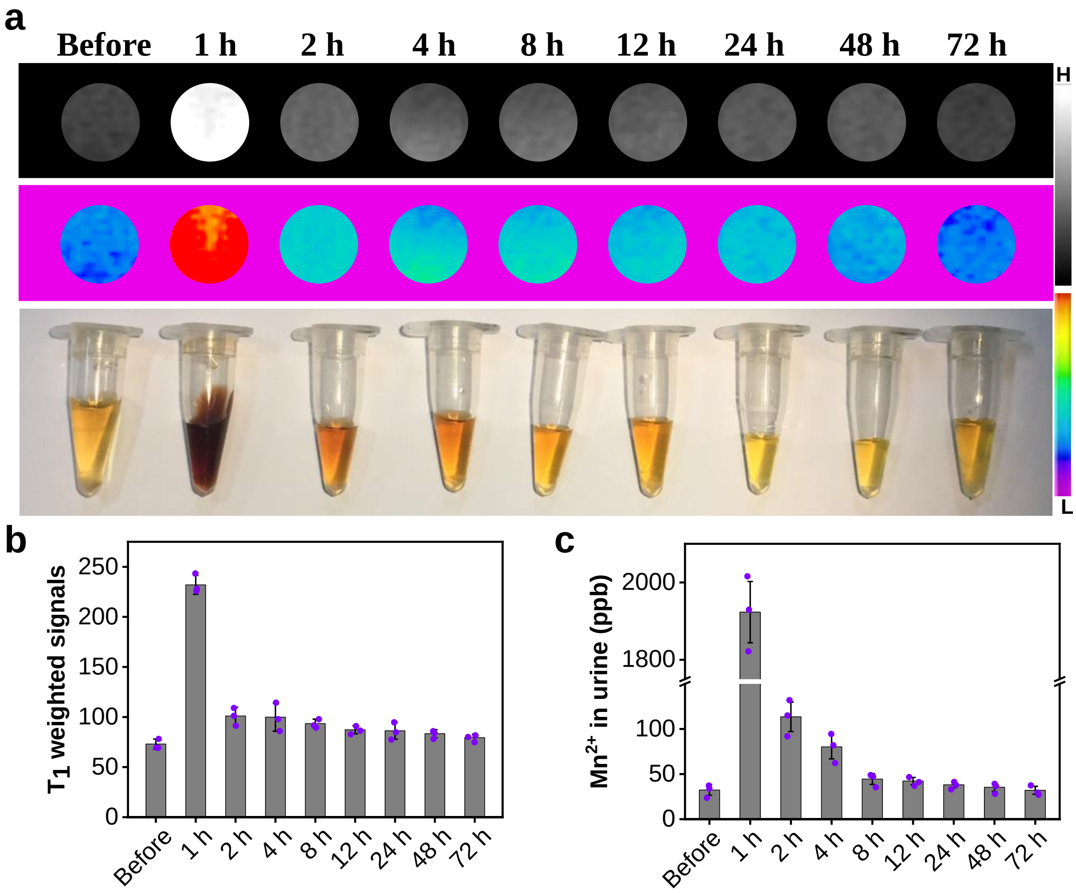

Supplementary Fig. 14. Renal clearance of Mn@CCs. (a) T_1-weighted MR images and Photograph of the urine of the healthy mice at different time points after intravenous administration of Mn@CCs. (b) Intensities of the urine of the healthy mice at different time points after intravenous administration of Mn@CCs (n=3 biologically independent samples). All the statistical data are expressed as mean values±SD. (c) The Mn^{2+} in the urine at different time points after intravenous administration of Mn@CCs (n=3 biologically independent samples). All the statistical data are expressed as mean values±SD.

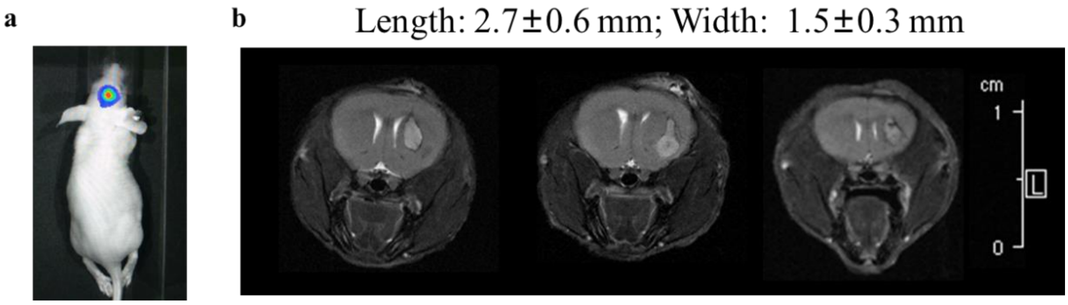

Supplementary Fig. 15. Successful establishment of U87MG glioma-bearing mice. (a) In vivo MR images of U87MG-bearing glioma mice at different time points after intravenous administration of Mn-CDs. (b) Relative signal change of brain at different time points based on MR imaging results.

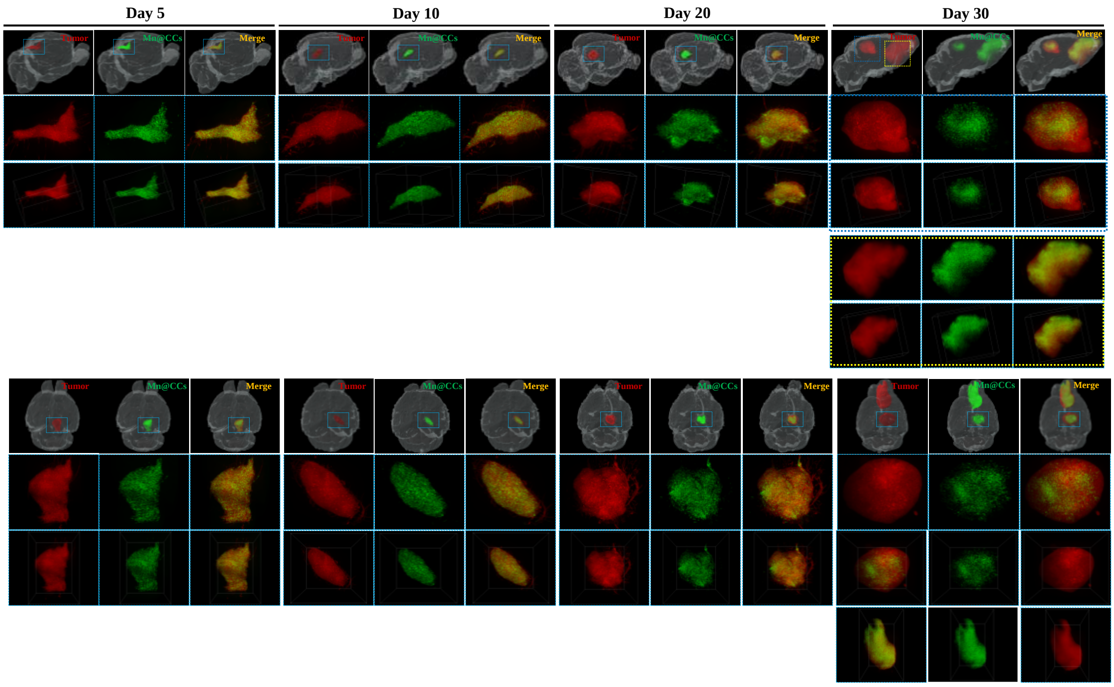

Supplementary Fig. 16. Wide-field images of whole brain from orthotopic glioma tumor-bearing mice at Day 5, Day 10, Day 20 and Day 30. Gray: brain tissue, red: RFP-expressed glioma, Green: Mn@CCs.

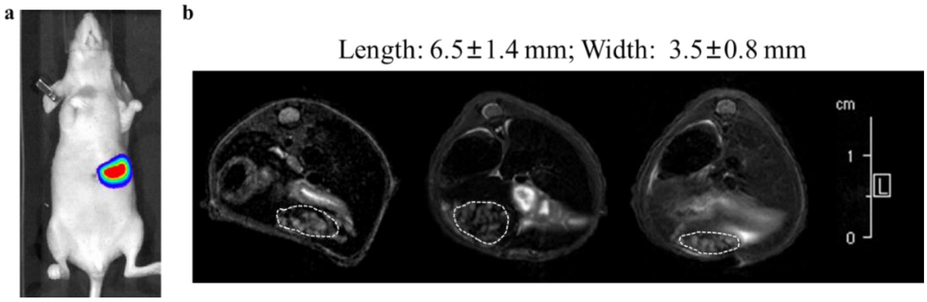

Supplementary Fig. 17. Successful establishment of HepG2-bearing mice. (a) Bioluminescence and (b) MRI were used to verify the successful establishment of HepG2-bearing mice.

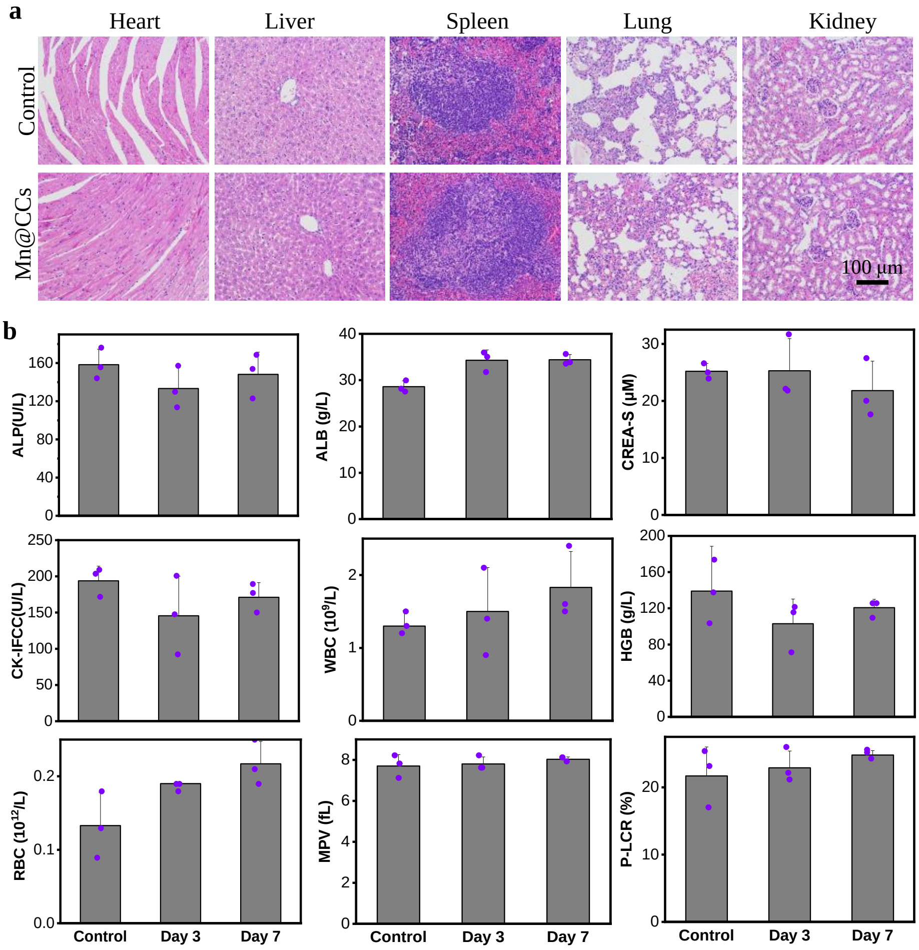

Supplementary Fig. 18. Evaluation of long-term biocompatibility of Mn@CCs. (a) H&E staining of different tissues after Mn@CCs treatment. Experiments were repeated three times. (b) Serum chemistry and hematological analysis of mice treated with Mn@CCs (n=3 biologically independent samples). All the statistical data are expressed as mean values±SD.
