# Supplementary material for: Carbonized paramagnetic complexes of Mn (II) as contrast agents for precise magnetic resonance imaging of sub-millimeter-sized orthotopic tumors
Source: Nat Commun. 2022 Apr 11;13:1938. doi: 10.1038/s41467-022-29586-w (PMC9001709; doi:10.1038/s41467-022-29586-w)
Supplement: Supplementary file 3 — Description of Additional Supplementary Files [file 41467_2022_29586_MOESM3_ESM.pdf]

Description of Additional Supplementary Files

File name: Supplementary Movie 1
Description: Related to Figure 3 / Observation of the Mn@CCs taken up by U87MG cells.

File name: Supplementary Movie 2
Description: Related to Figure 4 / The clearly whole process of Mn@CCs crossing the intact BBB.

File name: Supplementary Movie 3
Description: Related to Figure 7 / Three-dimensional (3D) render the tumors' morphology at different stages.
