# Supplementary figures and images for: Carbonized paramagnetic complexes of Mn (II) as contrast agents for precise magnetic resonance imaging of sub-millimeter-sized orthotopic tumors
Source: Nat Commun. 2022 Apr 11;13:1938. doi: 10.1038/s41467-022-29586-w (PMC9001709; doi:10.1038/s41467-022-29586-w)

## Slide 1
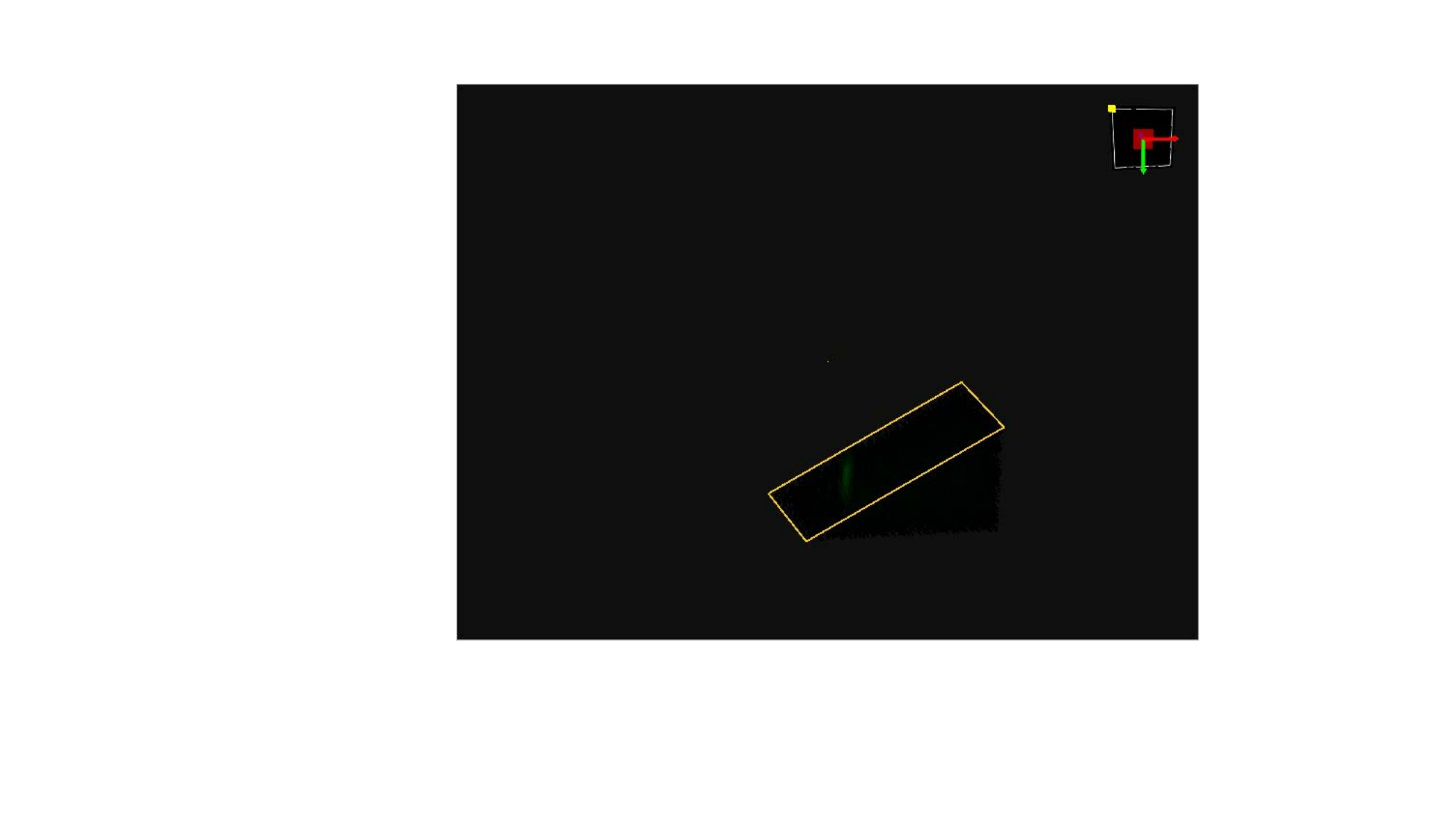

Supplement: Supplementary file 4 — Supplementary Movie1 [file 41467_2022_29586_MOESM4_ESM.pptx]

## Slide 1
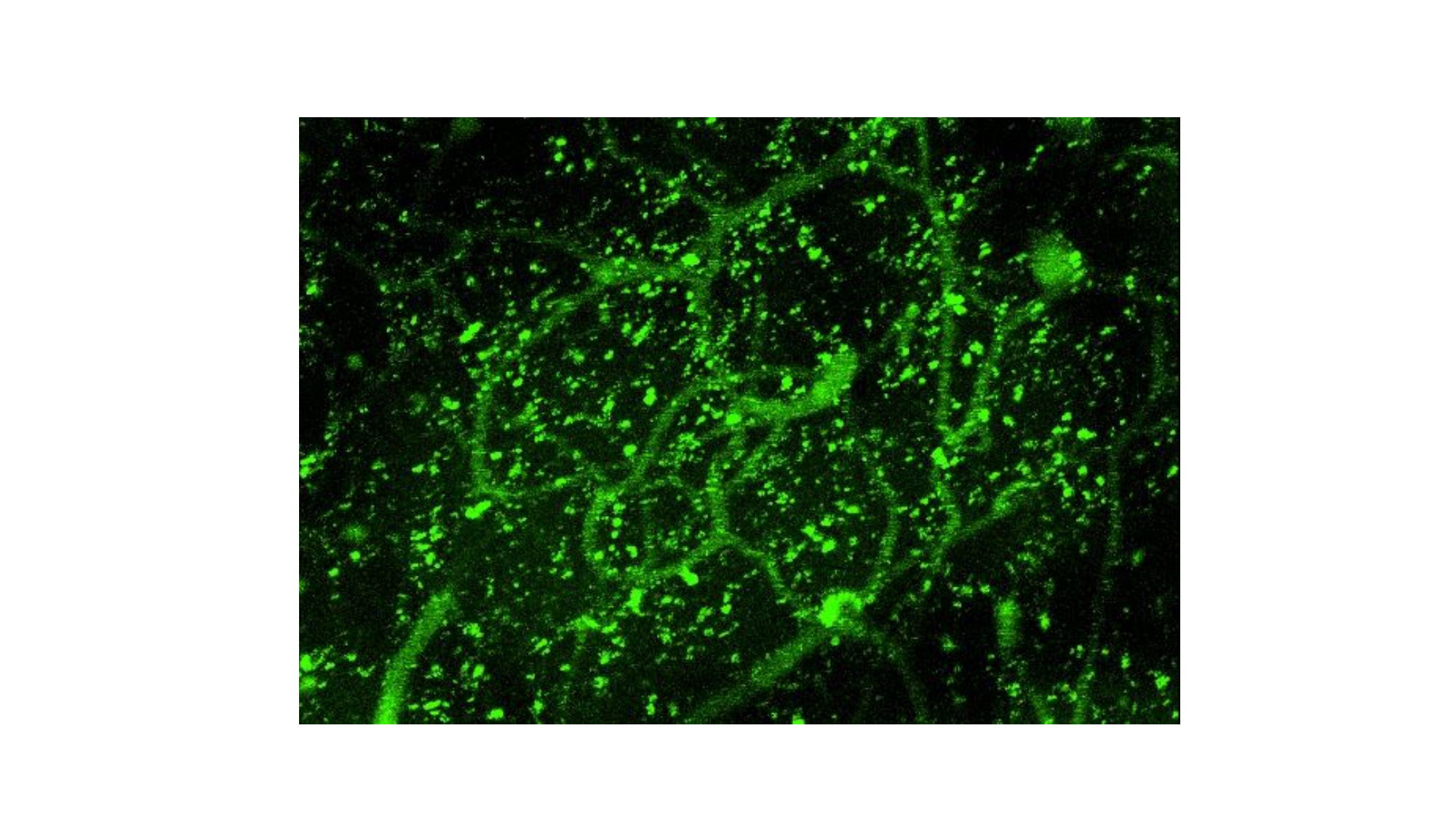

Supplement: Supplementary file 5 — Supplementary Movie2 [file 41467_2022_29586_MOESM5_ESM.pptx]
