# Supplementary material for: Carbonized paramagnetic complexes of Mn (II) as contrast agents for precise magnetic resonance imaging of sub-millimeter-sized orthotopic tumors
Source: Nat Commun. 2022 Apr 11;13:1938. doi: 10.1038/s41467-022-29586-w (PMC9001709; doi:10.1038/s41467-022-29586-w)
Supplement: Supplementary file 6 — Supplementary Movie 3 [file 41467_2022_29586_MOESM6_ESM.pptx]

## Slide 1
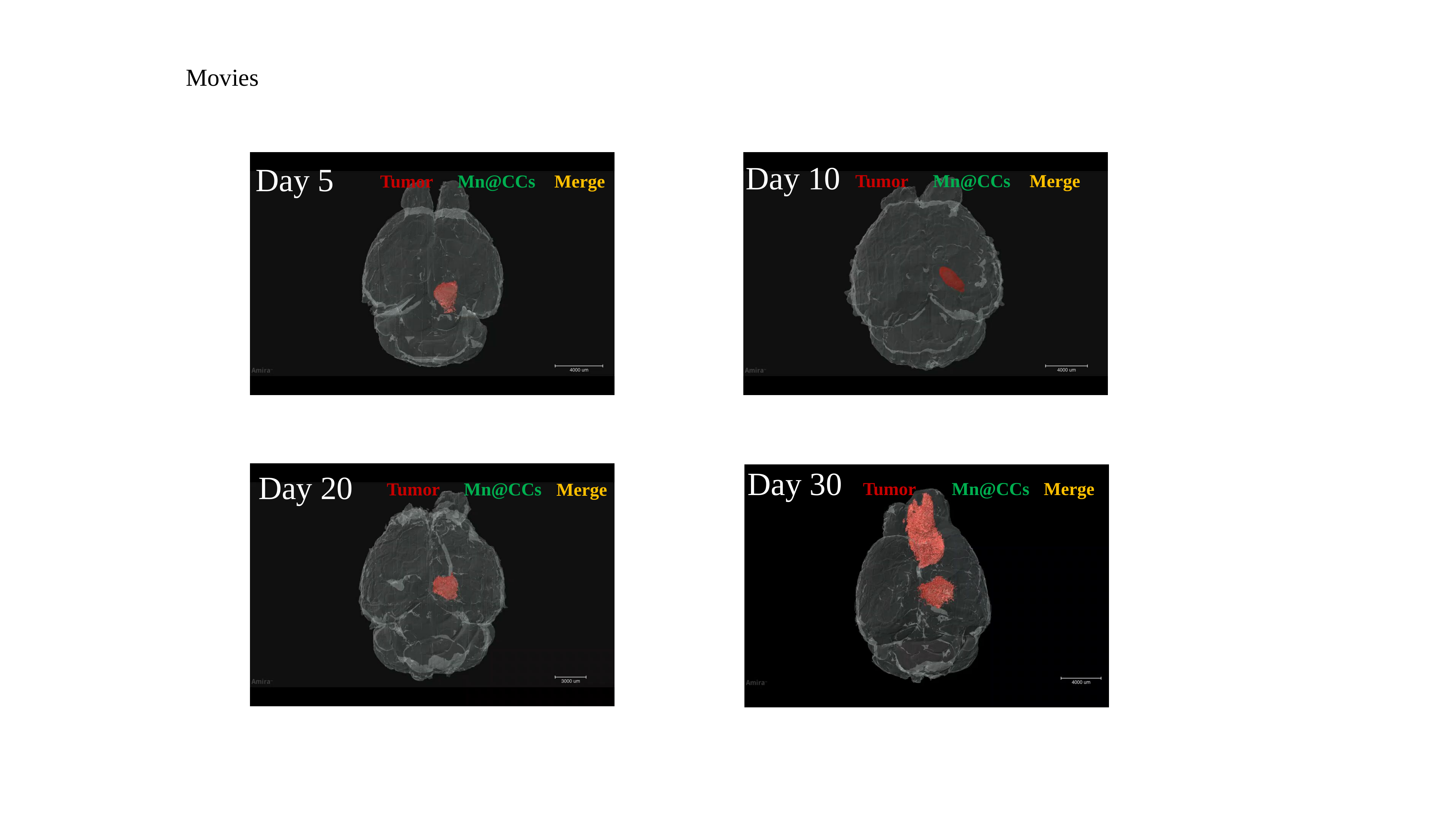

Movies
Day 10
Tumor
Mn@CCs
Merge
Day 5
Tumor
Mn@CCs
Merge
Day 30
Tumor
Mn@CCs
Merge
Day 20
Tumor
Mn@CCs
Merge
