# Supplementary material for: Carbonized paramagnetic complexes of Mn (II) as contrast agents for precise magnetic resonance imaging of sub-millimeter-sized orthotopic tumors
Source: Nat Commun. 2022 Apr 11;13:1938. doi: 10.1038/s41467-022-29586-w (PMC9001709; doi:10.1038/s41467-022-29586-w)
Supplement: Supplementary file 7 — Reporting Summary [file 41467_2022_29586_MOESM7_ESM.pdf]

nature research

Corresponding author(s): Chen, Hongmin

Last updated by author(s): Feb. 10, 2022

Reporting Summary

Nature Research wishes to improve the reproducibility of the work that we publish. This form provides structure for consistency and transparency in reporting. For further information on Nature Research policies, see our Editorial Policies and the Editorial Policy Checklist.

Statistics

For all statistical analyses, confirm that the following items are present in the figure legend, table legend, main text, or Methods section.

n/a	Confirmed	
☐	☒	The exact sample size (*n*) for each experimental group/condition, given as a discrete number and unit of measurement
☐	☒	A statement on whether measurements were taken from distinct samples or whether the same sample was measured repeatedly
☐	☒	The statistical test(s) used AND whether they are one- or two-sided<br>*Only common tests should be described solely by name; describe more complex techniques in the Methods section.*
☒	☐	A description of all covariates tested
☒	☐	A description of any assumptions or corrections, such as tests of normality and adjustment for multiple comparisons
☐	☒	A full description of the statistical parameters including central tendency (e.g. means) or other basic estimates (e.g. regression coefficient) AND variation (e.g. standard deviation) or associated estimates of uncertainty (e.g. confidence intervals)
☐	☒	For null hypothesis testing, the test statistic (e.g. *F*, *t*, *r*) with confidence intervals, effect sizes, degrees of freedom and *P* value noted<br>*Give P values as exact values whenever suitable.*
☒	☐	For Bayesian analysis, information on the choice of priors and Markov chain Monte Carlo settings
☒	☐	For hierarchical and complex designs, identification of the appropriate level for tests and full reporting of outcomes
☒	☐	Estimates of effect sizes (e.g. Cohen's *d*, Pearson's *r*), indicating how they were calculated

Our web collection on statistics for biologists contains articles on many of the points above.

Software and code

Policy information about availability of computer code

Data collection	Transmission electron microscopy (TEM) images were obtained by a FEI Tecnai G2 F30 Twin transmission electron microscope with a working voltage of 300 kV.<br>X-ray diffraction (XRD) pattern was obtained using Bruker D8 Advance X-ray diffractometer.<br>X-ray photoelectron spectroscopy (XPS) analyses were carried out with a K-Alpha+ X-ray Photoelectron Spectroscopy using a monochromatic Al Kα source (6 mA, 12 kV)(Thermo fisher Scientific).<br>The Raman spectrum was recorded using a laser RM2000 laser Raman spectrometer by excitation of 785 nm.<br>The Fourier transform infrared spectrum was obtained on a Nicolet is10 Infrared spectrometer.<br>Dynamic light scattering (DLS) and zeta potential were measured using Zetasizer Nano particle analyser series (Malvern Ltd., UK).<br>Thermogravimetric analysis (TGA) was performed by SDT Q600 (TA Instruments Co).<br>The UV-vis absorption spectra were collected on a cary60 PC spectrophotometer.<br>The fluorescence spectra were measured on an F-4600 spectrofluorometer.<br>Flow cytometry data were collected using Quanteon/ACEA (ACEA Biosciences) and the equipped NovoExpress for windows (version 1.5.6).<br>The fluorescence images of cells were imaged using an Olympus confocal microscope (Leica Microsystems, Germany).<br>The in vivo and ex vivo fluorescence imaging was conducted by IVIS Lumina II in vivo imaging system.<br>The T1-weighted MRI was performed on a 9.4 T BioSpec MRI (Bruker, Germany).<br>Evaluation in vivo the BBB-crossing ability of Mn@CCs were acquired using Olympus FVMPE-RS multiphoton laser scanning microscope.<br>Inductively coupled plasma mass spectrometry (ICP-MS) measurements were performed on PerkinElmer NexION 2000 ICP-MS.<br>3D fluorescent imaging of clearing tissue were acquired with Nuohai LS18 light sheet microscopy (Nuohai Life Science (Shanghai) Co, Ltd. All raw image data were collected in a dcimg format by LS 18 Nobelium software (v1.0.4) and those tiling of multiple position image stacks were processed into 16-bit 3D tiff format data using LS 18 Image Combine software (v1.0.1).
Data analysis	General data analysis: Origin 9.1 and 2018 (64-bit) SR1-b.9.6.1.195, GraphPad Prism 6.0, and software Image J2x V2.1.4.7. Quantitative results were presented as mean ± standard deviation.<br>MRI data analysis and processing: (1) T1-weighted MR (1.5T) images were acquired by a spin-echo (SE) sequence: TR (repetition time)/TE

(echo time) = 100/8.3 ms (T1), 128 × 128 matrices; (2) T1-weighted MR (7T) images: TR/TE = 300/10 ms (T1), 128 × 256 matrices; (3) T1-weighted MR (9.4T) images:TR/TE = 300/10 ms (T1), 128 × 256 matrices. The relaxivities were further analyzed by Origin 9.1.
3D MRI data analysis and processing: 3D MRI were acquired on a 9.4 T MRI system (Bruke 9.4T MicroMRI) with echo time=8.5 ms, effective TE =8.5 ms, number of experiments=8, repetition time=1000 ms, flip angle=180, rare factor=4, number of repetitions=1, number of averages= 6, matrix=256×256, FOV Read = 3 cm, slice thickness = 0.5 cm, slices = 50. 3D MR imaging reconstruction images and tumor volume calculation were generated using Amira 2020.1 (Thermo Fisher Scientific, USA) and Varian Eclipse Treatment Planning System (15.6).
3D fluorescent imaging of whole brain were acquired with Nuohai LS18 light sheet microscopy (Nuohai Life Science (Shanghai) Co., Ltd. At 6.3× zoom effective magnification, each sample was scanned by 6-tiles light-sheets axially at a 2.5 µm Z-step size. Fluorescent signals of Mn@CCs and tumors (U87 MG-RFP) were obtained by excitation at both 488 nm and 561 nm, respectively. All raw image data were collected in a dcimg format by LS 18 Nobelium software (v1.0.4) and those tiling of multiple position image stacks were processed into 16-bit 3D tiff format data using LS 18 Image Combine software (v1.0.1). Imaging processing and 3D rendering was performed with a Dell Precision 7820 workstation with Xeon Gold 5118 processor, 192 GB RAM and NVIDIA Quadro P4000. 3D reconstruction images were generated using Amira 2020.1 (Thermo Fisher Scientific, USA).
All the statistical data are expressed as mean ± SD. Statistical significance was assessed via a one-way ANOVA with Duncan post-hoc test and an unpaired two-tailed Student's t-test. The P value was analyzed by SPSS 22.0 (Statistical Package for the Social Sciences).

For manuscripts utilizing custom algorithms or software that are central to the research but not yet described in published literature, software must be made available to editors and reviewers. We strongly encourage code deposition in a community repository (e.g. GitHub). See the Nature Research guidelines for submitting code & software for further information.

Data

Policy information about availability of data

All manuscripts must include a data availability statement. This statement should provide the following information, where applicable:

- Accession codes, unique identifiers, or web links for publicly available datasets
- A list of figures that have associated raw data
- A description of any restrictions on data availability

The authors declare that all data related to this study are available in the article/and or its Supporting Information files and Source Data. Source data are provided with this paper.

Field-specific reporting

Please select the one below that is the best fit for your research. If you are not sure, read the appropriate sections before making your selection.

☒ Life sciences ☐ Behavioural & social sciences ☐ Ecological, evolutionary & environmental sciences

For a reference copy of the document with all sections, see nature.com/documents/nr-reporting-summary-flat.pdf

Life sciences study design

All studies must disclose on these points even when the disclosure is negative.

Sample size	No statistical method was used to predetermine the sample size for each study. For property measurement experiments, samples were prepared and tested three times independently. In vitro studies were repeated three times independently; For in vivo studies, each group contains at least 3 (n = 3-6) biologically independent samples/animals/independent experiments for evaluating the statistical significance.
Data exclusions	No data was excluded from the analysis.
Replication	For property measurement experiments, samples were replicated and tested independently for 3 times and after analysis the standard deviation was displayed. For each experiment the statistical analysis is indicated in the figure legends.
Randomization	Mice imaging study were randomized grouped by simple random sampling strategy.
Blinding	The investigators were not blinded to allocation during experiments and outcome assessment since our data analysis are based on objectively measurable data.

Reporting for specific materials, systems and methods

We require information from authors about some types of materials, experimental systems and methods used in many studies. Here, indicate whether each material, system or method listed is relevant to your study. If you are not sure if a list item applies to your research, read the appropriate section before selecting a response.

Materials & experimental systems

n/a	Involved in the study
☒	☐ Antibodies
☐	☒ Eukaryotic cell lines
☒	☐ Palaeontology and archaeology
☐	☒ Animals and other organisms
☒	☐ Human research participants
☒	☐ Clinical data
☒	☐ Dual use research of concern

Methods

n/a	Involved in the study
☒	☐ ChIP-seq
☐	☒ Flow cytometry
☒	☐ MRI-based neuroimaging

Eukaryotic cell lines

Policy information about cell lines

Cell line source(s)	U87MG, HepG2, NIH/3T3 and LO2 cell lines were purchased from Sigma-Aldrich (USA). U87MG-RFP cell line was purchased from Shanghai Zhong Qiao Xin Zhou Biotechnology Co. Ltd.
Authentication	Cell lines have been authenticated by short tandem repeat profiling, and the results were compared with reference database.
Mycoplasma contamination	All cell lines tested negative for mycoplasma contamination.
Commonly misidentified lines (See ICLAC register)	None.

Animals and other organisms

Policy information about studies involving animals; ARRIVE guidelines recommended for reporting animal research

Laboratory animals	Male BALB/c nude mice were purchased from Shanghai SLAC Laboratory Animal Co. Ltd (Shanghai, China) at the age of around 5-6 weeks after birth and housed in 12 light/12 dark cycle, 65-75°F (18-23°C), 40-60% humidity condition.
Wild animals	This study did not involve wild animals.
Field-collected samples	This study did not involve sample collected from the field.
Ethics oversight	All animal experiments were performed under a protocol approved by the Institutional Animal Care and Use Committee of Xiamen University. Animals were euthanized when the tumor reached 1.5 cm in diameter or when they became moribund with severe weight loss.

Note that full information on the approval of the study protocol must also be provided in the manuscript.

Flow Cytometry

Plots

Confirm that:

☒ The axis labels state the marker and fluorochrome used (e.g. CD4-FITC).

☒ The axis scales are clearly visible. Include numbers along axes only for bottom left plot of group (a 'group' is an analysis of identical markers).

☒ All plots are contour plots with outliers or pseudocolor plots.

☒ A numerical value for number of cells or percentage (with statistics) is provided.

Methodology

Sample preparation	U87 MG cells were seeded in a 24 well plate at a density of 5×104 cells/well. Cells untreated with Mn@CCs were denoted as the negative control group, while cells incubated only with Mn@CCs (50 μg mL-1) at 37 °C were denoted as the positive control group. To study energy-dependent processes, the cells were first preincubated at 4 °C instead of 37 °C for 1 h. After that, nanoparticles were added at a concentration of 50 μg/mL.The mixtures were incubation for another 4 h at 4 °C. Finally, the cells were trypsinized, washed and re-suspended in 0.5 mL PBS in tubes for flow cytometer (FCM) analysis. To analyze the different endocytotic uptake mechanisms, the cells were first preincubated with inhibitions in serum-free media for 1 h at 37 °C: NaN3 (60 mM), sucrose (450 mM), nystain (180 nM), and dynasore (80 μM). After that, Mn@CCs (50 μg mL-1) were added and coincubated with inhibitors for 4 h at 37 °C. Finally, the cells were trypsinized, washed and re-suspended in 0.5 mL PBS in tubes for FCM analysis.
Instrument	Flow cytometry data were collected using Quanteon/ACEA (ACEA Biosciences) and the equipped NovoExpress for windows version 1.5.6.

Software	Flow cytometry data were analyzed using NovoExpress for windows (version 1.5.6).
Cell population abundance	The absolute cells were analyzed for fluorescent intensity in the defined gate. The purity of post-sort fractions is regularly measured by the software. The fractions were around 20-40%.
Gating strategy	A forward-scatter (FSC)/side-scatter(SSC) gate was used to gate on U87 MG cells to exclude debris. FSC-H vs FSC-A gate was used to gated on U87MG singlet cells. Then, gates were set for Mn@CCs-positive using samples from PBS-treated controls (negative control). The detailed gating strategy could be found in Supplementary Fig. 9c.

☒ Tick this box to confirm that a figure exemplifying the gating strategy is provided in the Supplementary Information.
